# P-1969. Hepatotoxicity Signals Associated with Commonly Prescribed Oral Antibiotics: Insights from the FAERS Database

**DOI:** 10.1093/ofid/ofaf695.2136

**Published:** 2026-01-11

**Authors:** Albin C Sebastian, Linta Susan Kuriakose, Alvin Sunny

**Affiliations:** Square Hospital, West Panthapath, Dhaka, Bangladesh; Square Hospital, West Panthapath, Dhaka, Bangladesh; Square Hospital, West Panthapath, Dhaka, Bangladesh

## Abstract

**Background:**

Hepatotoxicity is a well-recognized but often underreported adverse effect of several commonly used oral antibiotics. While some hepatotoxic risks are described in pre-marketing trials, post-marketing surveillance is crucial to detect rare, delayed, or severe hepatic events. This study aimed to identify and quantify signals of antibiotic-associated hepatotoxicity using data from the U.S. FDA Adverse Event Reporting System (FAERS).

**Figure:**
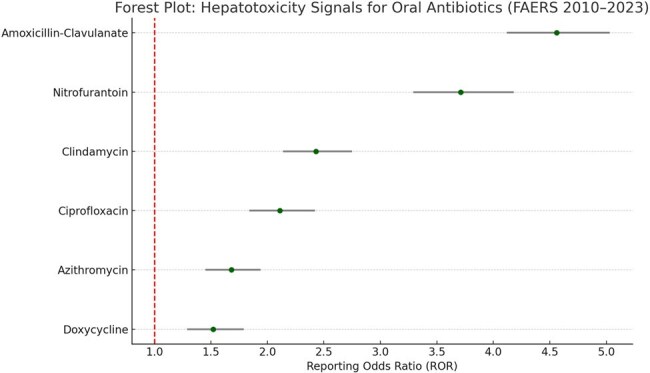
Forest Plot: Hepatotoxicity Signals for Oral Antibiotics (FAERS 2010–2023)

This forest plot presents the Reporting Odds Ratios (RORs) and 95% confidence intervals for hepatic adverse events associated with commonly prescribed oral antibiotics. Amoxicillin-clavulanate and nitrofurantoin showed the strongest disproportionality signals, particularly for cholestatic and hepatocellular injury, respectively. The findings underscore the importance of hepatic monitoring in patients prescribed these agents, especially in real-world, high-risk populations.

**Methods:**

A retrospective disproportionality analysis was conducted using FAERS data from January 2010 to December 2023. Reports of hepatobiliary adverse events were extracted using MedDRA Preferred Terms, including “drug-induced liver injury,” “hepatitis,” “cholestasis,” and “elevated liver enzymes.” The analysis focused on commonly prescribed oral antibiotics, including amoxicillin-clavulanate, azithromycin, ciprofloxacin, doxycycline, nitrofurantoin, and clindamycin. Reporting odds ratios (RORs) and 95% confidence intervals (CIs) were calculated to assess the strength of association, with a signal defined as ROR lower CI >1 and ≥3 cases.

**Results:**

A total of 9,328 hepatic adverse events were linked to the selected antibiotics. The strongest signal was observed for amoxicillin-clavulanate (ROR: 4.56; 95% CI: 4.12–5.03), followed by nitrofurantoin (ROR: 3.71; 95% CI: 3.29–4.18). Clindamycin (ROR: 2.43) and ciprofloxacin (ROR: 2.11) also showed significant associations. Azithromycin and doxycycline demonstrated weaker but notable signals. Hepatotoxicity types varied across agents, with cholestatic hepatitis being most common for amoxicillin-clavulanate, and hepatocellular injury more prominent in nitrofurantoin.

**Conclusion:**

This FAERS-based analysis highlights the differential hepatotoxicity profiles of widely used oral antibiotics. Amoxicillin-clavulanate and nitrofurantoin carry the strongest post-marketing hepatic safety signals. These findings support the need for liver function monitoring in high-risk patients and emphasize the value of pharmacovigilance systems in detecting real-world safety trends.

**Disclosures:**

All Authors: No reported disclosures

